# Hydrocephalus Internus

**Published:** 1850-06

**Authors:** Clinton Colegrove

**Affiliations:** Sardinia


ART. III. — *Hydrocephalus Internus. Chronic Diarrhœa.* By CLINTON COLEGROVE M. D.

DR. FLINT: — The following transcript is perhaps hardly interesting enough to merit publication, but trustful that its insertion may help to stimulate a more liberal contribution to your excellent Journal, I put it at your disposal. Its want of commanding points will be pardoned, if it should prove a suggestive impost on some laborious contemporary's dusty diary.

" *March* 11, 1850 — I have been latterly rather skeptical concerning the advantage of a professional diary, or note book, notwithstanding the almost universal testimony to its utility. I have doubted whether in reality, the record of cases would avail to impress their peculiar features more vividly and lastingly on the mind. And modernly, so numerous are the records and annotations of medical men, it cannot reasonably be expected, except in rare instances, that the medical world will derive particular benefit from the minute labors of their own ranks, since their name is "legion." Yet as an agreeable relaxation in the interims of toil, and as possibly a source of retrospective satisfaction, I will record perhaps the more striking and important cases with whose management, I am entrusted, either directly or incidentally.

I was called to see a child of Perry Hardy, and *en route*, Mr. Hardy, sen., requested me to call and see a sick girl at his house. Immediately I saw her. I concluded her symptoms grave, if not alarming. She had severe pain in her head, extending also to her back. She was continually complaining, uneasy, impatient of interrogation or manipulation, and inclined to somnolency or coma. Pulse 100, small and compressible. Countenance *pale*, though there was perceptible throbbing of the carotid arteries, and synchronous vibration of the head. She had been seized on the Friday or Saturday previous, four days having elapsed since the onset of the attack. The disease was ushered in by vomiting, head-ache, and exceeding irritability of the system. Constitution of the patient, scrofulous; temperament, nervous.

"Shall I bleed?" This question, almost as soon as it occurred, I answered negatively, but, as I afterwards thought, incorrectly. I was dissuaded from the adoption of this measure, from the *paleness* of the countenance, smallness and feebleness of the pulse, temperament of the patient, as well as from a lurking suspicion of the possibility of mistaking an active nervous disorder for inflammation of the brain. How then *did* I diagnose the disease? I answer, I did not *positively* diagnose it at first. But I ventured to pronounce it an attack of accute hydrocephalus. The age of the patient (12) might at first seem to contradict my opinion, but not absolutely. The rapidity of the progress of the case, also would seemingly invalidate this conclusion. Prescribed a dose of calomel and aloes, and small doses of calomel and James' Powder every three hours. The succeeding day revealed a decided change for the worse, and I regarded the case so hopeless as to hesitate about making any prescription.— But, deeming inaction at least culpable, I could not remain idle. Patient was now insensible, although screaming violently. Eyes staring and fixed, pupils dilated, countenance hippocratic, with strabismus. Hydrocephalus internus, is sometimes treated as synonymous with *arachnitis*. The uneasiness and irritability of the patient, conjoined with the violence of the pain, indicated, to me, membranous inflammation. Doubtless effusion of water had now occurred. I observed what is characteristic of hydrocephalus, an obstinate inclination of the head backwards against the pillow, as well as a disposition to *lie flat in bed*. Prescribed blisters to the calves of the legs—also Dover's powder with antimony and calomel in minute doses. If I erred at all in the treatment of this case, it was, I think, in withholding venesection, at my first visit. Inasmuch as this is pronounced the *sine qua non* in cerebral inflammation, I regretted that I did not pursue it, although I question its utility in the present instance. Treatment should have been earlier sought. She sunk at 3 P. M. of this day.

*March* 21. About two weeks since, I was requested to see Dea. S. Hudson, aged 75, of nervous temperament, and very spare habit. He had long suffered from a harassing cough, although his general health had been usually pretty good. Since August last, he has suffered almost incessantly from diarrhœa. He says he has had repeatedly, 15 or 20 dejections *per diem*. These were of a mucous character. This constant drain had emaciated and reduced him till he scarcely ventured from his house. When I saw him, he complained of anorexia, debility, œdema of the feet and general *malaise*. In accordance with the recommendation of Dr. Lee, I prescribed Nit. Arg. 3 grs. to the oz., and directed 5 minims to be taken thrice *per diem*, increasing the dose gradually, according to the effect produced. I advised this extreme caution both from the susceptibility to medicinal impressions on the part of the patient, and from an innate suspicion of the insecurity of largely prescribing an article so powerful, aside from the ill-advisedness of too suddenly checking a chronic discharge. It will be seen that patient got $\frac{1}{8}$th gr. a day. Prescibed also 3 grs. pulvis Dover's, at bed time every day. I heard little of my patient until yesterday,— when calling, I was delighted to learn that the discharge was completely arrested, and a *perfect cure* accomplished, at least so far as such a result could be secured in so short a space. Patient said—" I'm as regular as ever I was in the world." I must confess I did not anticipate a result so auspicious. Appetite considerably improved. I should have said, that for six months last previous, a slight interruption of the accustomed flux, exacerbates his cough, so that they uniformly alternated. But with his diarrhœa, his cough had also abated considerably. In short, I considered this a signal triumph of medicine over disease — and as every body had anticipated the deacon's gradual decline, the sudden disappearance of his disease will be considered, perhaps, marvellous. I am inclined to pronounce a *minuter* dose than the books and journals recommend, the safest and best. Dr. Chapman has recommended its prescription to an extent I should not dare to adopt abruptly.

The first of these cases appears to me interesting as exemplifying the occasional difficulty of discriminating between hydrocephalus and encephalitis. There was an evident intermingling of symptoms proper to both affections, and I cannot help suspecting that this is much oftener the case than from systematic treatises we might infer. Indeed, what would be *a priori*, more natural than to expect the coincidence of membranous and cerebral inflammation in the same case? It also illustrates the embarrassment which may sometimes befall the practitioner, in deciding one point, viz., venesection. The advanced period of the disease, with the circumstances already specified, dissuaded me from adopting a measure Herculean for weal or woe, and about the propriety of which I am still doubtful.

Yours, truly, C. COLEGROVE.

*Sardinia, April*, 16, 1850.

